# Concise Review: Stem Cells As an Emerging Platform for Antibody Therapy of Cancer

**DOI:** 10.1002/stem.513

**Published:** 2010-11

**Authors:** Richard T Frank, Joseph Najbauer, Karen S Aboody

**Affiliations:** a Department of Neurosciences, City of Hope National Medical Center and Beckman Research InstituteDuarte, California, USA; b Irell and Manella Graduate School of Biological Sciences, City of Hope National Medical Center and Beckman Research InstituteDuarte, California, USA; c Division of Neurosurgery, City of Hope National Medical Center and Beckman Research InstituteDuarte, California, USA

**Keywords:** Antibody, Blood-brain barrier, Cancer therapy, α-Carcinoembryonic antigen, CD3, Diabody, EGFR, HER2, Neural stem cells, Mesenchymal stem cells, scFv

## Abstract

Monoclonal antibodies are important tools for cancer therapy, however, three factors limit their effectiveness: toxicity, poor tumor penetration, and inability to cross the blood-brain barrier. This review discusses the emerging field of stem cell-mediated antibody delivery and how this approach may improve antibody therapy of cancer by overcoming these obstacles. STEM CELLS 2010;28:2084–2087

## INTRODUCTION

Recombinant monoclonal antibodies have emerged as important tools for cancer therapy. Despite the therapeutic advantages conferred by antibodies, three major weaknesses limit their effectiveness, that is, (a) poor penetration of solid tumors, (b) inability to cross the blood-brain barrier (BBB), and (c) mechanism-dependent and mechanism-independent toxicities [1,2]. Considerable research has focused on these obstacles and several novel solutions have been proposed to overcome them. However, no single solution has yet been able to address all three weaknesses.

Current methods require repeated systemic delivery of large quantities of antibodies to maintain therapeutic concentrations at tumor sites. However, an emerging strategy is the use of stem cells for in vivo antibody production. The inherent tumor-tropic properties of stem cells and their ability to traverse the BBB can be harnessed for tumor-selective delivery of such therapeutic antibodies. Using this approach, cumulative stem cell-mediated secretion of small quantities of antibodies specifically at tumor sites would be expected to result in therapeutically effective antibody concentrations. This approach could simultaneously overcome all three limitations of current antibody delivery methods and enhance therapeutic efficacy, while minimizing undesired exposure of healthy tissue to antibodies, thereby reducing side effects such as cardiac toxicity associated with the HER2/neu (human epidermal growth factor receptor 2)-specific antibody trastuzumab (Herceptin) (Genentech, South San Francisco, CA; http://www.herceptin.com/index.jsp) in some patients [1].

### Stem Cell Tropism to Tumors

Neural stem/progenitor cells (NSCs) and mesenchymal stem cells (MSCs) are multipotent cells that have an inherent ability to migrate to malignant tumor sites both within and outside of the central nervous system. Therapeutic benefit has been demonstrated with various anticancer agents, including interleukins, interferons, and prodrug-activating enzymes [3]. The molecular mediators of this tumor tropism include cytokines, growth factors, extracellular matrix (ECM), and ECM-remodeling proteins [4,5]. Unlike systemically delivered drugs, NSCs have been shown to infiltrate solid tumor parenchyma and also localize to hypoxic regions of tumors [6]. This provides a distribution advantage over intravenously (i.v.) delivered antibodies, which diffuse only in the immediate vicinity of blood vessels. Additionally, NSCs and MSCs have the ability to traverse the BBB to reach primary brain tumors or solid tumor metastases in the brain [3]. These advantageous properties suggest that stem cell-mediated delivery of antibodies may enable distribution to sites that are not readily accessible by i.v. injected antibodies and improve the effectiveness of cancer immunotherapy. Recent research has begun to investigate the full potential of using stem cells as a platform for antibody therapy.

### Stem Cell-Mediated Antibody Delivery

**Figure 1:**
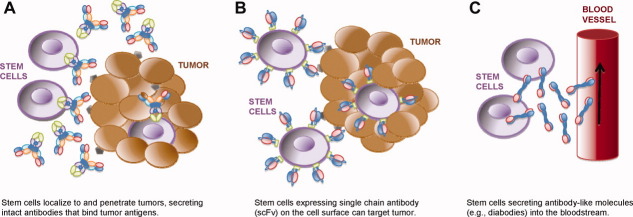
Strategies for stem cell-based antibody therapy of cancer. Models of potential strategies for the use of stem cells in antibody therapy. Delivery of antibodies or antibody-like molecules directly to tumor by tumor-tropic stem cells expressing either secreted antibody **(A)** or cell surface-bound antibody **(B)**. Use of stem cells as “biological pumps” to secrete antibodies or antibody-like molecules into the bloodstream **(C)**. All strategies utilize unique properties of stem cells to overcome limitations of traditional antibody therapy.

Stem cells can be genetically modified by viral and nonviral methods to express intact antibody or single-chain antibody fragments, such as scFv (single-chain variable fragment). Recently, it has been demonstrated that human NSCs can be transduced with adenoviral or lentiviral vectors encoding the heavy and light chains of anti-HER2 antibody [7] (Fig. 1A). NSCs secreted properly assembled anti-HER2 antibody, which specifically bound tumor cells and inhibited the proliferation of HER2 overexpressing breast cancer cells in vitro. Furthermore, i.v.-administered NSCs delivered anti-HER2 antibody to intramammary human breast cancer xenografts in immunodeficient mice. Importantly, anti-HER2 antibody was not detectable in the blood, whereas i.v. injected anti-HER2 antibody (trastuzumab) was present at high concentrations in both tumor and blood. This suggests that NSC-mediated antibody delivery may provide more specific tumor localization of therapeutic antibodies than i.v. injection of purified antibody, thereby potentially reducing associated toxicities to healthy tissues.

MSCs have also been investigated as vehicles for antibody delivery. Human MSCs have been nucleofected to express a cell surface-bound single chain antibody (scFv) targeting the glioma-associated epidermal growth factor receptor variant III (EGFRvIII) [8,9]. These scFv-expressing human MSCs localized to human glioma xenografts. In addition, the presence of scFv enhanced the retention of MSCs in the tumor parenchyma [9] (Fig. 1B). Exposure of glioma xenografts in the mouse flank to anti-EGFRvIII scFv-bearing MSCs resulted in a 50% reduction in tumor size. Furthermore, in an intracranial human glioma xenograft model, coinjection of scFv-expressing MSCs with glioma cells significantly improved the survival of experimental mice. Reduced vascularization of tumors in mice receiving coinjection of MSCs was also observed, indicating that MSCs may have an additional therapeutic benefit of reducing tumor angiogenesis [9].

### Stem Cells As “Biological Pumps” for Therapeutic Antibodies

In addition to the use of stem cells for tumor-specific antibody delivery, a recent study has explored the use of MSCs as biological pumps to secrete antibody fragments into the circulatory system to achieve sustained therapeutic effect. Human MSCs expressing bispecific diabodies (single chain antibody fragments) targeting both α-carcinoembryonic antigen (α-CEA) and the T cell CD3 receptor persisted for more than 40 days when implanted subcutaneously in immunodeficient mice [10] (Fig. 1C). Furthermore, secreted diabody was detectable in mouse blood for more than 6 weeks, far exceeding the serum half-life of injected purified diabody [10,11]. MSC-secreted diabodies activated tumor-specific T cells and reduced tumor burden in mice bearing α-CEA-expressing colon cancer xenografts [10].

### Considerations for Stem Cell-Mediated Antibody Therapy

Factors that must be considered when evaluating stem cells as a platform for antibody therapy include: (a) potential immunogenicity of stem cells, (b) the optimal stem cell lineage, (c) the preferred source of stem cells, and (d) whether this approach is capable of achieving therapeutic concentrations of antibody at the tumor sites.

#### Immunogenicity of Stem Cells

Cellular therapies, including stem cells must overcome immunological barriers to transplantation, and the potential for stem cell immunogenicity has been the subject of controversy. Several findings suggest that stem cells may possess some degree of “immune privilege” that protects against immune rejection. This unique immune status has been reported for embryonic stem cells, NSCs, and MSCs. However, recent studies have uncovered evidence that casts doubt on such immune privilege. Both NSCs and MSCs under noninflammatory conditions do not express MHC (major histocompatibility complex) class II antigens and express only low levels of MHC class I and costimulatory molecules, such as CD80 and CD86 [12,13]. This makes it unlikely that such stem cells would be directly targeted by cytolytic T cells. Despite low resting levels of MHC class I, NSCs are not lysed by natural killer (NK) cells, which typically target cells lacking MHC class I [14]. NSCs and MSCs produce anti-inflammatory cytokines, including (transforming growth factor beta), which have been shown to inhibit T cell-mediated response to stem cells [12,15]. Studies examining the transplantation of NSCs and MSCs in laboratory animals have yielded differing results. For example, mouse NSC allografts were not rejected from recipient mice, unless mice were presensitized with donor mouse splenocytes [16]. Human NSCs implanted into immunocompetent mice bearing syngeneic glioma persisted for at least 2–3 weeks with only subacute, localized T cell infiltrate without administration of immunosuppressive drugs (Aboody et al., unpublished data). Wennersten et al. reported poor NSC survival in a rat model of stroke, however, a brief regimen of the immunosuppressive drug cyclosporin A significantly improved the survival of NSCs, and this tolerance persisted at least 6 months after the cessation of immunosuppression [17]. Therefore, stem cell survival is also influenced by the immediate microenvironment in which they are implanted. Other strategies to enhance stem cell survival after transplantation include modification of stem cells to increase resistance to apoptosis by overexpressing Act [18,19], heme oxygenase-1 expression [20], or downregulation of MHC class I expression on stem cells by viral stealth mechanism [21]. Together, these data suggest that the hypoimmunogenic properties of NSCs and MSCs may be insufficient to prevent their rejection by an immunocompetent host, however, immunosuppressive drugs, as well as genetic manipulation of stem cells may allow such stem cells to persist long enough to mediate a therapeutic effect.

#### Stem Cell Lineage

The first major consideration in developing stem cells for therapeutic applications is to identify the type of stem cell that is best suited for antibody therapy. In addition to NSCs and MSCs, many other types of stem cells are potentially suitable for this approach. Ultimately, the choice of stem cell will depend on tumor tropism, efficiency of antibody production and post-translational modifications, immunogenicity, and safety.

If delivery of antibody to tumors is the desired application, robust tumor tropism of stem cells is crucial. Although NSCs and MSCs both display tumor tropism in vivo, their exact mechanisms of tumor tropism do not necessarily overlap. As a result, one of these stem cell types may demonstrate superior tropism to a particular tumor, and therefore, prescreening of a patient's tumor could potentially be used to determine the type of stem cell that would display the highest tumor tropism.

Another factor that may favor one stem cell lineage over another is the cell's capacity to produce effective antibodies. We have demonstrated that NSCs can secrete intact antibody [7], but it is possible that other types of stem cells, such as MSCs, may be able to sustain higher levels of antibody production. In addition to the quantity of antibody produced, another important consideration is antibody potency. The glycosylation profile of an antibody significantly affects its ability to induce effector functions such as antibody-dependent cellular cytotoxicity and complement-dependent cytotoxicity [2]. The optimal stem cell for antibody synthesis would produce antibodies with glycosylation profiles that favor enhanced effector functions. The glycosylation of antibodies produced by NSCs and MSCs has not yet been characterized, but is an important subject for future study.

Finally, MSCs have been reported to have multiple safety concerns, including contributing to tumor stroma and facilitating tumor allografts in mice [22,23]. Furthermore, a recent study demonstrated that bone marrow-derived MSCs can enhance the metastatic potential of a weakly metastatic human breast cancer cell line in mice [22]. Such safety concerns for MSCs and other types of stem cells can potentially be mitigated by introducing suicide genes into the therapeutic stem cells (e.g., HSV-Tk, *E. coli* cytosine deaminase, or carboxylesterase) [3,24], which may allow elimination of the stem cells at the end of the antibody therapy. Nevertheless, these findings demonstrate the need for careful investigation of stem cell and tumor interactions prior to the application of stem cell-mediated antibody therapy in human patients.

#### Source of Stem Cells

In addition to stem cell lineage, consideration should also be given to the optimal source of stem cells. Potential options include cells derived from autologous, allogeneic, or xenogeneic sources. Autologous stem cells are patient-derived and have the advantage of being nonimmunogenic, giving them the potential to persist longer in vivo. However, a disadvantage of autologous stem cells is that depending on the type of stem cell desired, they may be quite difficult to isolate and expand in sufficient quantities. NSCs, for example, are significantly harder to isolate than are bone marrow- or adipose tissue-derived MSCs. Induced pluripotent stem cells (iPSCs) may provide an additional source of autologous stem cells [25], but, to our knowledge, no studies have yet investigated the tumor targeting or antibody expression abilities of iPSCs, although their potential warrants such investigations. Allogeneic stem cells are derived from a nongenetically identical human donor, and use of allogeneic stem cells may facilitate the establishment of “off-the-shelf” stem cell lines that would be available to a greater number of patients. The potential of stem cells to display immune privilege, as discussed earlier, might allow allogeneic stem cells to resist immune rejection long enough to be therapeutically effective. Xenogeneic cells derived from mouse or other species are another potential cell source, but these cells are the least likely to survive immune rejection and may carry additional safety concerns.

Stem cell-mediated therapy will require large numbers of cells. Primary cells, however, have a limited capacity for ex vivo propagation and expansion, largely due to differentiation in culture, which leaves little time for genetic manipulation to induce antibody expression. Bulk cultures of cells are also difficult to characterize because of inherent heterogeneity. Stem cells immortalized with v-*myc*, human telomerase, SV40 large T-antigen, or other methods provide a means to maintain and expand clonal stem cell lines in culture indefinitely. In addition, immortalized clonal cell lines are more likely to be stable and can be extensively characterized. However, use of oncogenes to induce immortalization carries safety concerns that must be adequately addressed before such cells can be used clinically. To minimize safety concerns, immortalized cell lines can be engineered to express suicide genes, such as cytosine deaminase or HSV-Tk, to facilitate their elimination [3].

#### Concentration of Antibody at Tumor Site

A final consideration is whether stem cell-mediated antibody delivery can generate a therapeutically effective concentration of antibody at the tumor site. Tumor-localized antibody production is expected to require significantly less antibody to attain therapeutic concentrations at the tumor site than systemic administration of antibodies. However, whether even this concentration can be achieved is not yet known. Factors influencing the concentration of antibody at the tumor site include: (a) the number of stem cells reaching the tumor, (b) the tumor volume covered by stem cells, (c) the amount of antibody produced per stem cell, (d) the duration of stem cell persistence at the tumor site, and (e) antibody pharmacokinetics. The number of stem cells reaching the tumor will depend, at least in part, on the number of cells delivered, strength of tumor tropism and the route of administration. Our data from glioma xenograft models indicate that intracranially injected NSCs can achieve 70%–90% tumor coverage, which may be sufficient to elicit a therapeutic effect [26]. The quantity of antibody produced by stem cells will depend on multiple factors, including the vectors and expression strategy used. Stem cell fate over time must also be determined for each disease model. The pharmacokinetic properties of the antibody, including tumor uptake and clearance, will be determined by the molecular size and composition of the antibody (e.g., intact, scFv, and diabody) [11]. All of these factors will require optimization to achieve maximal therapeutic efficacy.

## CONCLUSION

The use of antibodies for cancer therapy has brought positive clinical outcomes for many patients, however, some limitations remain. Stem cell-mediated antibody therapy could facilitate sustained release of antibody specifically at the tumor site, allowing therapeutic concentrations to be achieved without the need for repeated systemic administration of high doses of antibody. Thus, application of stem cell-mediated antibody delivery has the potential to reduce toxicities associated with systematically administered antibodies. The success of this approach for cancer therapy depends on the ability of the antibody-secreting cells to reach tumor foci and to persist long enough to achieve a therapeutic effect. The tumor-tropic properties of stem cells and their unique ability to cross the BBB make them promising delivery vehicles for therapeutic antibodies. Additionally, the use of stem cells as biological pumps may allow sustained release of therapeutic molecules into the bloodstream. No single therapeutic modality can be expected to be effective against all cancers, and this will likely also be the case for stem cell-based cancer therapies. Nevertheless, leveraging the unique properties of stem cells to overcome current limitations has the potential to greatly expand the power of antibody therapy to combat cancer.

## DISCLOSURE OF POTENTIAL CONFLICTS OF INTEREST

The authors indicate no potential conflict of interest.

## References

[1] Adams GP, Weiner LM (2005). Monoclonal antibody therapy of cancer. Nat Biotechnol.

[2] Carter PJ (2006). Potent antibody therapeutics by design. Nat Rev Immunol.

[3] Aboody KS, Najbauer J, Danks MK (2008). Stem and progenitor cell-mediated tumor selective gene therapy. Gene Ther.

[4] Najbauer J, Danks MK, Schmidt NO, Bertolotti R, Ozawa K (2007). Neural stem cell-mediated therapy of primary and metastatic solid tumors. Progress in Gene Therapy, Autologous and Cancer Stem Cell Gene Therapy.

[5] Kendall SE, Najbauer J, Johnston HF (2008). Neural stem cell targeting of glioma is dependent on phosphoinositide 3-kinase signaling. Stem Cells.

[6] Zhao D, Najbauer J, Garcia E (2008). Neural stem cell tropism to glioma: Critical role of tumor hypoxia. Mol Cancer Res.

[7] Frank RT, Edmiston M, Kendall SE (2009). Neural stem cells as a novel platform for tumor-specific delivery of therapeutic antibodies. Plos One.

[8] Balyasnikova IV, Franco-Gou R, Mathis JM (2010). Genetic modification of mesenchymal stem cells to express a single-chain antibody against EGFRvIII on the cell surface. J Tissue Eng Regen Med.

[9] Balyasnikova IV, Ferguson SD, Sengupta S (2010). Mesenchymal stem cells modified with a single-chain antibody against EGFRvIII successfully inhibit the growth of human xenograft malignant glioma. Plos One.

[10] Compte M, Cuesta AM, Sanchez-Martin D (2009). Tumor immunotherapy using gene-modified human mesenchymal stem cells loaded into synthetic extracellular matrix scaffolds. Stem Cells.

[11] Wu AM, Senter PD (2005). Arming antibodies: Prospects and challenges for immunoconjugates. Nat Biotechnol.

[12] Ubiali F, Nava S, Nessi V (2007). Allorecognition of human neural stem cells by peripheral blood lymphocytes despite low expression of MHC molecules: Role of TGF-beta in modulating proliferation. Int Immunol.

[13] Uccelli A, Moretta L, Pistoia V (2008). Mesenchymal stem cells in health and disease. Nat Rev Immunol.

[14] Mammolenti M, Gajavelli S, Tsoulfas P (2004). Absence of major histocompatibility complex class I on neural stem cells does not permit natural killer cell killing and prevents recognition by alloreactive cytotoxic T lymphocytes in vitro. Stem Cells.

[15] Patel SA, Meyer JR, Greco SJ (2010). Mesenchymal stem cells protect breast cancer cells through regulatory T cells: Role of mesenchymal stem cell-derived TGF-beta. J Immunol.

[16] Hori J, Ng TF, Shatos M (2003). Neural progenitor cells lack immunogenicity and resist destruction as allografts. Stem Cells.

[17] Wennersten A, Holmin S, Al Nimer F (2006). Sustained survival of xenografted human neural stem/progenitor cells in experimental brain trauma despite discontinuation of immunosuppression. Exp Neurol.

[18] Mangi AA, Noiseux N, Kong D (2003). Mesenchymal stem cells modified with Akt prevent remodeling and restore performance of infarcted hearts. Nat Med.

[19] Lee HJ, Kim MK, Kim HJ (2009). Human neural stem cells genetically modified to overexpress Akt1 provide neuroprotection and functional improvement in mouse stroke model. Plos One.

[20] Tang YL, Tang Y, Zhang YC (2005). Improved graft mesenchymal stem cell survival in ischemic heart with a hypoxia-regulated heme oxygenase-1 vector. J Am Coll Cardiol.

[21] Lee EM, Kim JY, Cho BR (2005). Down-regulation of MHC class I expression in human neuronal stem cells using viral stealth mechanism. Biochem Biophys Res Commun.

[22] Mishra PJ, Mishra PJ, Glod JW (2009). Mesenchymal stem cells: Flip side of the coin. Cancer Res.

[23] Studeny M, Marini FC, Dembinski JL (2004). Mesenchymal stem cells: Potential precursors for tumor stroma and targeted-delivery vehicles for anticancer agents. J Natl Cancer Inst.

[24] Zischek C, Niess H, Ischenko I (2009). Targeting tumor stroma using engineered mesenchymal stem cells reduces the growth of pancreatic carcinoma. Ann Surg.

[25] Yamanaka S (2007). Strategies and new developments in the generation of patient-specific pluripotent stem cells. Cell Stem Cell.

[26] Lin D, Najbauer J (2007). Novel method for visualizing and modeling the spatial distribution of neural stem cells within intracranial glioma. Neuroimage.

